# Suicide among ambulance service staff: a review of coroner and employment records

**DOI:** 10.29045/14784726.2020.12.4.4.10

**Published:** 2020-03-01

**Authors:** Becky Mars, Kelly Hird, Fiona Bell, Cathryn James, David Gunnell

**Affiliations:** University of Bristol; University Hospitals Bristol NHS Foundation; Yorkshire Ambulance Service NHS Trust; Yorkshire Ambulance Service NHS Trust; Association of Ambulance Chief Executives; University of Bristol; University Hospitals Bristol NHS Foundation Trust

**Keywords:** ambulance staff, paramedics, suicide

## Abstract

**Background:**

There is growing evidence to suggest that ambulance service staff may be at increased risk for suicide; however, few studies have explored risk factors within this occupational group.

**Aim:**

To investigate factors commonly associated with ambulance staff suicides.

**Method:**

Eleven ambulance service trusts across the United Kingdom were asked to return details of staff suicides occurring between January 2014 and December 2015. Coroners were then contacted to request permission to review the records of the deceased.

**Results:**

Fifteen suicides were identified (73% male, mean age 42 years). Inquest data were available on 12 deaths. The most common method used was hanging. Possible risk factors identified included recent return to work following a period of sickness absence, poor mental health, relationship and debt problems, history of self-harm and the loss of a driving licence/change in job role.

**Conclusion:**

Identifying characteristics of suicide among this high-risk group is important to inform the development of suicide prevention initiatives. Additional research is needed with an adequate control group to further explore the risk factors identified in this study.

## Introduction

There are marked variations in suicide risk between different occupations. Healthcare professionals, particularly nurses, have been found to have an elevated risk compared to other occupational groups (Alderson, Parent-Rocheleau, & Mishara, 2015; Hawton, Agerbo, Simkin, Platt, & Mellanby, 2011; Meltzer, Griffiths, Brock, Rooney, & Jenkins, 2008; Office for National Statistics, 2017). Recent figures from the United Kingdom and Australia have demonstrated that ambulance personnel are also at increased risk (Milner, Witt, Maheen, & LaMontagne, 2017b; Office for National Statistics, 2017). For example, the risk of suicide among male paramedics in the United Kingdom was found to be 75% higher than the national average. (There were too few suicides among female paramedics to reliably estimate risk.) Moreover, among male health professionals (including dentists, doctors and nurses), paramedics were the only group that had evidence of a heightened suicide risk (Office for National Statistics, 2017). However, little is known about the factors that may influence risk among ambulance service (AS) staff.

A systematic review of research investigating suicide among first responders (police, firefighters, emergency medical technicians (EMTs) and paramedics) identified only two studies that focused on EMTs/paramedics (Stanley, Hom, & Joiner, 2016). Surveys of ambulance personnel conducted in several countries have revealed high levels of mental health problems and suicidal thoughts and attempts, and low levels of help-seeking among this group (Jones, 2017; Mind, 2015; Newland, Barber, Rose, & Young, 2015; Sterud, Hem, Lau, & Ekeberg, 2008). In a UK survey of over 1300 AS staff, problems at work were often cited as the main cause of mental health problems. These included excessive workload, pressure from management, long hours, changing shift patterns and exposure to traumatic incidents (Mind, 2015). Exposure to trauma – a situation frequently experienced by paramedics – has been linked with increased capability for suicide (i.e. the degree to which an individual feels able to make a suicide attempt), which is a key component of recent theoretical models of suicide causation (Klonsky & May, 2015; O’Connor, 2011; Van Orden et al., 2010). In line with this, a recent study found that the extent of exposure to suicide attempts and deaths was positively correlated with suicidal behaviour among firefighters (Kimbrel et al., 2016).

Despite being a high-risk occupational group, we are not aware of any studies that have investigated the characteristics of AS staff members who have died by suicide. This is important to inform prevention initiatives. The present study uses coroner records to identify characteristics and risk factors associated with suicide among ambulance staff in the United Kingdom.

## Methods

Eleven ambulance NHS trusts in the United Kingdom (10 in England and the Welsh Ambulance Service) were contacted regarding the study. Trusts in Scotland and Northern Ireland were not contacted as it would not have been feasible for the research team to travel to these locations within the funding constraints for this study. Trusts were asked to return details of deaths that had occurred between January 2014 and December 2015 where the coroner recorded a suicide, accident/misadventure, narrative (a longer explanation which sets out the facts surrounding the death in more detail) or open conclusion (where there is insufficient evidence for any other conclusion) (Crown Prosecution Service, 2017); the latter categories of death were included, as previous research suggests that these are often self-inflicted (Gunnell et al., 2013). ASs were also asked to return employment data including information on sickness absence, disciplinary record, job role and access to AS-provided well-being services. Eight ASs returned data relating to 15 members of staff. Throughout this article, we refer to these deaths as suicide but recognise that in some cases they may not have been given a suicide conclusion. Table 1 provides a breakdown of the information received from each AS trust.

**Table 1.** Summary of data availability.

Ambulance trust	Returned data on staff suicide	Number of staff suicides reported	Returned data on their well-being access	Returned data on their sickness record	Returned data on their disciplinary record	Returned data on their job role
1	Yes	1	No	Yes	Yes	Yes
2	Yes	3	No	No	No	Yes
3	Yes	3	Yes	Yes	Yes	Yes
4	Yes	4	Yes	Yes	Yes	Yes
5	Yes	1	No	Yes	Yes	Yes
6	Yes	1	Yes	No	Yes	Yes
7	Yes	1	No	Yes	Yes	Yes
8	Yes	1	Yes	Yes	Yes	Yes
9	(No suicides reported)	0	N/A	N/A	N/A	N/A
10	No	N/A	No	No	No	No
11	No	N/A	No	No	No	No

Permission to access inquest records was requested from the relevant coroner. Following approval, a researcher visited each coroner and completed a data extraction form (Supplementary 1) to record information on possible risk factors and precipitants. Data were collected on known risk factors for suicide, including: (a) demographics; (b) financial and job problems; (c) relationship problems; (d) coroner’s decision and cause of death; (e) psychiatric and medical history; and (f) alcohol and substance use. A detailed description (vignette) of the circumstances leading up to the death including any precipitating factors was also compiled. Details of deaths with a misadventure, narrative or open decision (n = 3) were independently reviewed by two of the study authors with expertise in suicide research (BM and DG) and rated according to the likelihood they were a suicide (based on the rater’s professional opinion). Two cases were rated as high likelihood of suicide and one was rated as low-moderate. Findings were similar when excluding the individual with a low-moderate rating.

## Results

### Demographics and employment information

Eleven of the 15 identified suicides were male (73%) and the mean age was 42 (range 22–55). The recorded ethnicity for all the deaths was White British. Nine staff members were confirmed to be in patient facing roles, three worked in the AS call centres (999/111 in the United Kingdom) and three were defined as ‘other’. All worked full-time hours and the mean length of service was 16 years (73% > 10 years). Four of the deceased had a job role/location change in the study period, of which two occurred in the six months prior to their death. These included moving to a different station/trust and changing the number of hours worked. The reason for these changes was not provided.

Occupational health (OH) data on access to well-being services were available for nine individuals (from four AS trusts). Four had accessed the OH department through their AS, four had not accessed the service and one person declined OH support. Eleven of the 15 AS staff had data available regarding sickness absence during the study period (January 2014–December 2015). Each of these staff members reported at least one episode of sickness (Table 2) (42 episodes in total). Reasons included mental health (n = 5, 12% of episodes), musculoskeletal problems (n = 5, 12% of episodes), other problems (includes colds, coughs and gastro-intestinal reasons) (n = 12, 29% of episodes) and unknown reasons (n = 20, 47% of episodes). Over half (6/11) of the sample died within one month of returning to work following a period of sickness absence. One staff member was on sickness leave at the time of their death. None of the individuals were suspended from work at the time of their deaths.

**Table 2.** Sickness episodes.

Individual	Number of sickness absences	Total number of sickness days
1	1	25
2	3	12
3	2	147
4	16	Missing
5	1	11
6	2	66
7	6	50
8	1	33
9	2	7
10	5	43
11	3	5

### Findings from coroner data

Eleven coroners allowed the researcher access to the deceased’s records and one forwarded a copy of the inquest report. Data are therefore available for 12 of the 15 deaths (80%) identified by the AS. Nine of the deceased were in patient-facing roles and three were call centre workers. Findings were similar when excluding the three call centre workers, therefore data are presented for the whole sample.

Data on demographics and risk factors are only available for individuals whose coroner’s records were reviewed (n = 11). The inquest record contained information on gender, occupation and the circumstances of the death. The age and gender distribution was similar to that of the whole sample. With regards to marital status, 27% (n = 3/11) were single, 36% (n = 4/11) were married and 36% (n = 4/11) were divorced/separated. Around a third (36%) were living alone at the time of their death. The largest proportion of deaths were due to hanging (n = 8/12, 66.7%). Other causes of death included poisoning, hypovolaemia and carbon monoxide toxicity. Nine (75%) of the deaths were recorded as a suicide, two were narrative and one was recorded as a misadventure. Three of the 12 deaths (20%) had morphine recorded in their toxicology report and this was identified as the cause of death in two cases. There was evidence to suggest alcohol had been consumed in approximately a third of cases (4/11).

Table 3 provides information about potential occupational and socioeconomic risk factors. Relationship problems, disciplinary problems at work and debt problems were each identified in 4/11 cases (n = 36%). Relationship problems included recent separation/relationship breakdown and falling out with a close friend. Two of the disciplinary issues were related to sickness absence. Debt problems ranged considerably from smaller payday and bank loans to very large amounts of debt. Loss of/restricted driving licence was also found to be a potential risk factor present in three of the deaths (27%). Problems with mental health were common (Table 4); 9/12 (75%) had a psychiatric disorder at the time of their death (most often depression/anxiety) and approximately a third (4/11) had a history of self-harm (defined as any intentional non-fatal self-poisoning or self-injury, irrespective of degree of suicidal intent or nature of other types of motive) (National Institute for Health and Care Excellence, 2012).

**Table 3.** Potential occupational and socioeconomic risk factors.

Risk factor	Sample N
Debt problems, n (%)	4/11 (36.4)
Relationship problems, n (%)	4/11 (36.4)
Disciplinary issues at work, n (%)	4/11 (36.4)
Loss of driving licence resulting in dismissal or change in job role, n (%)	3/11 (27.3)

Note: Total N = 11 as data are not known for one individual. Some individuals reported multiple risk factors.

**Table 4.** Psychiatric history.

Risk factor	Sample N
History of psychiatric services contact, n (%)	5/11 (45.5)
Psychiatric disorder at time of death *(yes/probably)*, n (%)^1^	9/12 (75.0)
Primary disorder at time of death, n (%)^1^ *Affective disorder (mainly depression/anxiety)* *Other disorder* *No disorder*	8/12 (66.7) 1/12 (8.3) 3/12 (25.0)
History of psychiatric disorder in the past, n (%)	7/11 (63.6)
Time of last GP contact, n (%) < 1 month before death < 6 months Unknown	6/11 (54.5) 3/11 (27.3) 2/11 (18.2)
Previous self-harm, n (%)^2^	4/11 (36.4)

Note: Total N = 11 as data are not known for one individual. Some individuals reported multiple risk factors.

^1^Data were reported on the inquest report so data on current psychiatric disorder are available for 12 individuals.

^2^Note there was also one interrupted suicide which is not included as a suicide attempt.

## Discussion

As far as we are aware, this is the first study to explore risk factors for suicide among AS staff. Fifteen deaths were identified over the two-year study period (January 2014–December 2015), although this is likely an underestimate of the total number of suicides of AS staff in England and Wales, as not all ASs returned data. Potential risk factors include recent return to work following sickness absence, relationship problems, psychiatric disorder, self-harm, debt problems and change in job role.

### Comparison with previous research

The characteristics of those who had died by suicide in this study are in line with national data, which indicates that the highest rates of suicide are found among males aged 45–59 (Office for National Statistics, 2016). A higher proportion of registered paramedics are male (62%) than female (Health and Care Professions Council, 2017), and in keeping with this, and the increased risk of suicide among males in the general population, nearly three quarters of the suicides identified in this sample were male. The mean age of death among the paramedic suicides is consistent with the age distribution of all paramedics, with the highest proportion of the workforce being aged between 40 and 49 (Health and Care Professions Council, 2017).

Risk factors for suicide (including self-harm and psychiatric disorder) among the paramedic deaths were similar to those reported in a recent review of coroners’ records of suicides regardless of occupation in England (Palmer et al., 2014). The high levels of mental health problems found in our study could be considered surprising given that the sample were all in full-time employment at the time of their death (less than 40% were employed in the study by Palmer et al., 2014). However, a number of recent surveys have found that mental health problems, suicidal thoughts and suicide attempts are common among AS staff (Jones, 2017; Mind, 2015; Newland et al., 2015; Sterud et al., 2008). While many of the risk factors for suicide among AS staff are in line with those of the general UK population, some factors were identified that may be more specific to the AS, such as loss of/restricted driving licence (although numbers were small). Given the nature of the work of a paramedic, a change in licence status would likely necessitate a change in job role, or even dismissal from the AS. It is noteworthy that 4/9 deaths with data available from employment records had a change in job role/location in the period before their death, although the reasons for this were not known and it is possible that the development of mental health problems may have led to the change in work pattern rather than them being causally related to suicide.

According to the occupational records, seven of those who died by suicide did so while on sickness absence, or within one month of returning to work (data available for 11 cases). In addition, two individuals were identified from the coroner records as having received disciplinary action due to prolonged sickness. Returning to work following a period of sickness appears to be an important risk factor identified in this study. Findings suggest that individuals should receive additional support during this time, particularly if the absence is prolonged or is related to mental health difficulties.

The majority of individuals in this sample killed themselves by hanging, which is the most common method used in the United Kingdom (Office for National Statistics, 2016). There is evidence to suggest that occupational based knowledge and access to lethal means may influence suicide rates (Milner, Witt, Maheen, & LaMontagne, 2017a). Morphine was identified as either the cause of death or a contributing factor in a third of the paramedic deaths (3/9). In the first case, the morphine was obtained via occupational access, in the second it was obtained through private employment outside of the NHS and in the third case information regarding the source was not available. While there was no evidence to suggest that ease of access to controlled drugs led to a higher proportion of deaths from poisoning than expected (Office for National Statistics, 2016), the use of morphine in each case is noteworthy, as this drug is not one which is commonly used in fatal poisonings in the United Kingdom. Restricting access to means is an effective strategy for suicide prevention (Sarchiapone, Mandelli, Iosue, Andrisano, & Roy, 2011). However, access to and knowledge of controlled drugs is integral to the job role of a paramedic, and so restricting access may not be a feasible strategy for suicide prevention in this group.

### Limitations

The findings need to be interpreted in light of several limitations. First, the total sample included was small, meaning findings need to be interpreted with caution. Second, there was variation in the depth and breadth of information included in the coroner’s records, and it is possible that some risk factors may not have been recorded. Third, the eligible sample was limited to those who were employed within an AS trust during the study period. Staff working in similar roles in other sectors (e.g. for private organisations) and those who had retired/changed professions were not included. Finally, for most of the risk factors we identified, data across the wider AS workforce were unavailable; such data would have enabled our findings to be put into context. However, the approach taken (reviewing risk factors among those who have died by suicide) has been used in many previous studies to explore heightened risk of suicide in different occupational groups (Conner, 2011; Hawton et al., 2002; Hawton, Malmberg, & Simkin, 2004; Malmberg, Simkin, & Hawton, 1999).

## Conclusion

Ambulance personnel are known to be at higher risk of suicide (Milner et al., 2017b; Office for National Statistics, 2017). This study identified several risk factors for suicide within this occupational group, including returning to work following a period of sickness absence, mental health, relationship and debt problems, history of self-harm and the loss of a driving licence/change in job role. Additional research with an adequate control group (i.e. AS staff who have not died by suicide) is needed to explore these issues further. Further research should also expand on this work by conducting psychological autopsy studies, and qualitative studies involving staff members who have attempted suicide, to identify relevant risk factors and explore attitudes towards help-seeking.

## Acknowledgements

We thank all the ambulance services that participated in the study. We also thank all members of the steering group for their contribution to this study: Will Hancock ((steering group chair) South Central Ambulance Service NHS Foundation Trust); David Davis (College of Paramedics); Kerry Gulliver (East Midlands Ambulance Service NHS Trust); Jean Hayes (The Ambulance Staff Charity); Alan Lofthouse (Unison); Julian Mark (Chair, National Ambulance Services Medical Directors Group (NASMeD)); Faye McGuinness (Mind); Anna Parry (Association of Ambulance Chief Executives); Sue Putman (South Central Ambulance Service NHS Foundation Trust); and Sarah Thompson (South Western Ambulance Service NHS Foundation Trust). We would also like to thank Simon Talbott who helped with the development of the study protocol.

## Conflict of interest

All authors have completed the ICMJE uniform disclosure format at www.icmje.org/coi_disclosure.pdf.

## Ethics

Ethical approval was granted on 20 May 2016: REC reference 16/NE/0148. HRA approval was granted on 20 May 2016: IRAS ID 202720. UKCRN ID: 31046.

## Funding

The research was funded by the Association for Ambulance Chief Executives (AACE).
